# Identification of risk factors and development of a multivariable prognostic prediction model for incontinence-associated dermatitis in older nursing home residents

**DOI:** 10.1038/s41598-026-40416-7

**Published:** 2026-02-18

**Authors:** Monira El Genedy-Kalyoncu, Bettina Völzer, Jan Kottner

**Affiliations:** Charité – Universitätsmedizin Berlin, Corporate Member of Freie Universität Berlin and Humboldt-Universität zu Berlin, Institute of Clinical Nursing Science, Charitéplatz 1, 10117 Berlin, Germany

**Keywords:** Urinary incontinence, Fecal incontinence, Dermatitis, Prediction models, Risk factors, Long-term care, Prevention, Diseases, Health care, Medical research, Risk factors

## Abstract

Incontinence-associated dermatitis (IAD) is an inflammatory skin condition caused by prolonged exposure of the skin to urine and feces. It is common among older adults in long-term care and contributes to pain, discomfort, and increased risk for secondary infection. This study aimed to identify risk factors for IAD development and to construct a prognostic model for incontinent nursing home residents aged ≥ 65 years. A secondary analysis was conducted using data from a cluster-randomized controlled clinical trial performed in Berlin, Germany (April 2019 to June 2021). A total of 149 incontinent residents were included. At week 12, 20 residents (13.4%) newly developed IAD. The final cluster-adjusted multivariable model identified lower Barthel Index scores, severe mobility impairment, and the absence of xerosis on the legs as significant predictors. An interaction between double incontinence and Barthel Index scores indicated that the protective effect of higher functional independence was reduced in residents with double incontinence. The model demonstrated good discrimination (area under the curve 0.82), sensitivity of 78.6%, specificity of 74.2%, and satisfactory calibration (Hosmer-Lemeshow test *p* = 0.67). These findings support early risk identification and targeted prevention; validation in a prospective cohort is warranted to confirm the model’s robustness and clinical utility.

**Supplementary Information:**

The online version contains supplementary material available at 10.1038/s41598-026-40416-7.

## Introduction

According to the International Classification of Diseases, Eleventh Revision (ICD-11; code EK02.22), incontinence-associated dermatitis (IAD) is defined as “irritant contact dermatitis from prolonged contact with urine or faeces as a result of incontinence”^1^. IAD is a frequent inflammatory skin disorder in older adults and one of the most common skin conditions in long-term care^2,3^.

Clinically, IAD may present with erythema, maceration, vesicles, or nodular thickening, which may progress to the loss of superficial skin layers^4,5^. It primarily affects the genital, buttock, and upper thigh areas and can extend to the lower back, particularly in bedridden individuals or those experiencing substantial urinary or fecal leakage. Typical symptoms include itch, burning, and pain, and complications such as secondary infections or pressure ulcers^6,7^. These manifestations can cause discomfort, sleep disturbance, and reduced quality of life and may lead to considerable distress for affected individuals^5,8,9^. Moreover, IAD imposes a substantial burden on healthcare systems by increasing nursing workload, treatment costs, and the risk of further skin damage^10^.

The global population aged 65 years or older is rising rapidly, accompanied by increasing rates of frailty, chronic disease, and dependency^11,12^. Incontinence is among the most prevalent geriatric care problems, affecting up to 80% of residents in nursing homes^4,13,14^. Because incontinence exposes the skin to persistent moisture and irritants, these individuals are inherently at risk of developing IAD. In long-term care settings, IAD prevalence among incontinent individuals ranges from 3% to 23%, depending on case definitions and assessment methods^14–16^. Given demographic trends, the number of affected residents is expected to grow over the coming decades.

In this context, nurses hold a central role in maintaining skin integrity and preventing IAD through regular skin assessments, individualized continence management, and evidence-based cleansing and protection routines^17–19^. Effective nursing interventions can significantly influence skin health outcomes and reduce the overall care burden associated with IAD. Early identification of residents at risk is essential to initiate preventive measures and avoid progression to more severe skin damage^20^. Consequently, strategies that enhance early recognition and targeted prevention are highly relevant to daily nursing practice.

The pathophysiology of IAD is multifactorial, involving chemical irritation, friction, and prolonged moisture exposure^4^. A recent systematic review focusing on prognostic factors for IAD development identified, among others, fecal incontinence, immobility, and prolonged skin moisture as important risk factors^21^. However, their relative contribution remains uncertain due to methodological heterogeneity across studies. An expert consensus further highlighted moisture exposure, stool consistency, and reduced mobility as key determinants of IAD risk, particularly relevant in geriatric long-term-care populations^22^. Nevertheless, the strength and interaction of these factors are not yet well quantified.

Despite growing attention to these risk factors, robust prognostic evidence is limited. No validated prediction model currently exists to estimate individual IAD risk among older incontinent residents. Developing such tools is essential to guide targeted prevention, improve resource allocation, and distinguish modifiable from non-modifiable determinants of risk.

The objective of this study was to identify individual risk factors for IAD development and to develop a prognostic model for incontinent nursing home residents aged 65 years and older to predict those at highest risk.

## Methods

### Source of data

For this secondary analysis, data originating from an investigator-initiated, outcome-assessor-blinded, exploratory cluster-randomized controlled trial were used. The trial was conducted between April 2019 and June 2021 in 17 residential long-term care facilities in the federal state of Berlin, Germany and included 314 residents^23^. The primary objective of the underlying clinical trial was to assess the effects of implementing an evidence-based skincare program to prevent common adverse skin conditions in residential long-term geriatric care settings, including skin tears, pressure ulcers, IAD, xerosis cutis, and intertrigo. Ethical approval was granted by the ethics committee of Charité – Universitätsmedizin Berlin, Germany, (approval number: EA1/243/18), the trial was prospectively registered at ClinicalTrials.gov (Identifier: NCT03824886) and the German Clinical Trials Register (drks.de; Identifier: DRKS00028954). The trial adhered to the Declaration of Helsinki and its amendments, and the International Council for Harmonization guideline for Good Clinical Practice (ICH Topic E6 (R1)). Written informed consent was obtained from all participants or their legal representatives prior to study participation. The underlying clinical trial was funded by the German Federal Ministry of Education and Research (BMBF; grant number 01GL1801). A detailed study protocol and the main results of the trial have been published elsewhere^23,24^.

This study is reported in accordance with the TRIPOD for Clustered Data (TRIPOD-Cluster) extension for transparent reporting of multivariable prediction models developed using clustered datasets^25^.

### Sample selection and eligibility criteria

#### Participant-level eligibility:

(I) Permanent residency in the nursing home at baseline; (II) age ≥ 65 years; (III) substantial care-dependency according to the German Social Code Book XI (SGB XI; care level II or higher); (IV) written informed consent provided by the resident or legal representative. Due to ethical considerations, residents receiving end-of-life care were excluded. Residents requiring specialized dermatological treatment at baseline were also excluded to avoid interference between medical treatments and skincare interventions.

#### Cluster-level eligibility:

(I) Nursing homes located in the federal state of Berlin, Germany; (II) capacity of at least 70 beds; and (III) an institutional pressure ulcer prevention standard in place. Each nursing home constituted one cluster.

#### Dataset-level eligibility for analysis:

For this analysis, the full dataset of the underlying clinical trial was available without restrictions. All clusters and all participants from both the intervention and control groups were included, provided that they (I) met the participant/cluster-level eligibility criteria, (II) completed both the baseline and week 12 follow-up visit, (III) were incontinent at baseline (urinary and/or fecal), and (IV) showed no signs of IAD at baseline.

Trial results reported by Völzer et al. indicated minimal clustering of the outcome IAD across nursing homes (ICC = 0.03)^26^. Accordingly, data from intervention and control groups were pooled to enhance statistical precision and model stability. Group allocation was not included as a predictor in the primary model but was re-evaluated in sensitivity analyses to confirm the absence of bias^27^.

### Outcome

The outcome of interest was incident IAD at 12 weeks after baseline among residents with urinary and/or fecal incontinence. IAD was diagnosed and classified according to the Ghent Global Incontinence-Associated Dermatitis Categorisation Tool (GLOBIAD), assigning categories 1A, 1B, 2A or 2B^28,29^. Head-to-toe skin examinations were conducted by dermatologists at baseline and at the week 12 follow-up visit. An incident case was defined as any newly developed IAD of GLOBIAD category 1A, 1B, 2A, or 2B identified at the week 12 visit. All examiners were study physicians who had completed trial-specific training, were experienced in assessing older nursing home residents, and were blinded to group allocation. This standardized protocol ensured consistency and reliability in data collection. The prediction modeling was conceived post hoc; therefore, outcome assessors were unaware of any modeling aims at the time of data collection.

### Predictors

#### Data collection procedures

Baseline data were collected from primary and secondary sources prior to randomization using standardized case report forms. Demographic and health-related data were extracted from medical records and interviews with participants. For participants with cognitive limitations, missing information was obtained by interviewing relatives, legal representatives, or nursing staff. If data could not be obtained via this process, they were labelled and reported as missing. Data collection processes were uniformly conducted across all clusters. Data were subsequently entered into an electronic case report form. Data collection was exclusively performed by trained researchers, study nurses, and clinicians who were members of the study team.

#### Variables and operationalization

##### Demographic variables (collected at baseline):

Age [years] at the time of the baseline visit, sex [male/female], duration of residency [months] reflecting the time between admission to the nursing home and the baseline visit, Body Mass Index (BMI) was analyzed both as a continuous variable using the formula weight [kg]/height [m²] and as categorical variables: Overweight (BMI ≥ 25) and Underweight (BMI < 18.5), according to definition of the World Health Organization (WHO)^30^.

##### Cognitive and functional assessments (collected at baseline):

The Global Deterioration Scale (GDS) to measure the severity of cognitive impairment, ranging from 1 (no cognitive impairment) to 7 (very severe cognitive impairment)^31^. Barthel Index to measure physical function related to basic activities of daily living, ranging from 0 to 100, with higher scores indicating greater functional independence^32^. Braden Scale for pressure ulcer risk assessment, ranging from 6 to 23 points, where 18 or higher indicates low risk, 15–17 moderate risk, 13–14 high risk, and 12 or lower very high risk^33^. For the Barthel Index and the Braden Scale, both total scores and subscale scores were explored, but not entered simultaneously in multivariable models. Care level was classified according to the German Social Code Book XI^34^.

##### Health-related variables (collected at baseline):

Medical conditions were coded according to ICD-11^35^. The variable Dementia comprised all cases with ICD-11 codes 6D8*, which include Alzheimer’s disease and other dementia subtypes. Severe mobility impairment was operationalized as documented motor weakness or paresis, specifically defined by the presence of hemiparesis (ICD-11 6B60.6) or paralytic syndromes (e.g., tetra-/para-/hemiplegia) (ICD-11 codes MB5*).

##### Medication (collected at baseline):

Regular medication was classified according to the Anatomical Therapeutic Chemical (ATC) classification system and grouped into therapeutic subcategories, for example agents acting on the renin–angiotensin system (ATC code C09) or corticosteroids (ATC code H02)^36^. Polypharmacy was operationalized as the concurrent, regular use of at least five distinct medications on a daily basis^37^.

##### Skin Conditions (collected at baseline and week 12):

IAD (ICD-11 EK02.22) was categorized in accordance with GLOBIAD^28,29^. Dry skin (xerosis cutis; ICD-11 ED54) was categorized according to the Overall Dry Skin (ODS)^38^. Pressure ulcers (ICD-11 EH90) were categorized according to the National Pressure Ulcer Advisory Panel/European Pressure Ulcer Advisory Panel/Pan Pacific Pressure Injury Alliance (NPUAP/EPUAP/PPPIA) classification^39,40^, with only category II or higher considered. Intertrigo (ICD-11 EK02.2) was defined according to ICD-11 as an irritant contact dermatitis of skin folds resulting from friction, sweating or contact with body fluids^35^. Skin tears were categorized according to International Skin Tear Advisory Panel (ISTAP) (Type I–III)^41^. For all skin conditions, the presence or absence of the condition was considered as a binary variable (yes/no); location and severity were not included in this analysis. All candidate variables were analyzed in two ways: first, as single independent risk factors for the development of IAD, and second, as inputs to the multivariable prediction model.

### Sample size

For this secondary analysis, no formal sample size calculation was performed. However, the full dataset of the underlying clinical trial was available and used to maximize the number of outcome events and ensure stable parameter estimation. Therefore, all participants and clusters that met the inclusion criteria of this analysis were included without any restrictions. This approach ensured maximal use of all available data, increased the number of outcome events (newly developed IAD at week 12), and thereby enhanced the robustness of the analyses^27^.

### Statistical analysis methods

#### Descriptive analysis

Continuous/metric variables were described using means and standard deviations (SD) or medians and interquartile ranges (IQR; 25–75%). Categorical variables, including the outcome newly developed IAD 12 weeks after baseline, were presented as numbers (n) and proportions (%). Outcome and predictor data were complete for all residents included in the prediction analysis. No item-level missingness occurred, and no imputation was performed.

#### Predictor selection

Demographic, health-related, and functional variables were compared between incontinent residents who developed new IAD at week 12 and those who remained free of IAD. Continuous variables were analyzed using mean differences (MD), and categorical variables using odds ratios (ORs) with 95% confidence intervals (CIs). Group differences with *p* ≤ 0.05 (two-sided), ORs ≤ 0.5 or ≥ 2, or notable MDs were considered as potential predictors. Univariate binary logistic regressions were then performed to estimate associations with IAD. Variables with *p* ≤ 0.05 were considered statistically significant and included as candidate predictors. Additionally, clinically relevant variables (e.g., based on feasibility in practice or prior evidence) were retained irrespective of statistical significance.

Pairwise associations between candidate predictors were examined to identify collinearity. Pearson correlation coefficients were calculated for continuous variables, point-biserial correlations for continuous–dichotomous pairs, and Chi-square tests with Phi coefficients for dichotomous pairs. Pairs with |r| > 0.8 or with strong conceptual overlap were considered redundant. To avoid collinearity, measures of the same construct (e.g., BMI and its categories, Braden and Barthel total and subscores) were never entered simultaneously. Depending on statistical performance and theoretical relevance, either the total score or a specific subscore was retained.

#### Model derivation

All candidate predictors identified after univariate screening and collinearity assessment were initially entered simultaneously into a multivariable logistic regression model (ENTER method). Exploratory stepwise procedures based on the Wald statistic (backward, forward, bidirectional) were performed as sensitivity analyses to evaluate statistical robustness of predictor retention. The final specification was determined manually, combining these exploratory results with clinical considerations of relevance and feasibility, to ensure that predictors were both statistically stable and applicable in practice.

Multicollinearity in the final model was assessed using variance inflation factors (VIF), with all values < 5, supporting the absence of problematic collinearity.

#### Internal validation and performance assessment

To account for potential overfitting, internal validation was performed with 1,000 bias-corrected and accelerated bootstrap resamples. The model’s discriminatory ability was quantified using the Area Under the Curve (AUC) of the Receiver Operating Characteristic (ROC) curve. AUC values range from 0.5 (no discrimination) to 1.0 (perfect discrimination), with values above 0.7 generally considered acceptable and values above 0.8 indicative of good discrimination.

Sensitivity (proportion of true positives correctly identified) and specificity (proportion of true negatives correctly identified) were calculated at predefined probability cutoffs (0.10, 0.30, 0.50, 0.70) and at an additional balanced cutoff identified through grid search. This approach allowed evaluation of performance across different decision thresholds, including a cut point with approximately balanced sensitivity and specificity.

Calibration was evaluated both numerically and graphically. Calibration-in-the-large (average difference between predicted and observed risk) and the calibration slope (ideal value = 1) were calculated. A calibration plot compared predicted and observed event rates across probability strata. As global measures, the Hosmer-Lemeshow test (*p* > 0.05 indicating adequate fit) and the Brier score (lower values indicating higher overall accuracy) were also reported.

#### Sensitivity analyses

To assess robustness, several additional analyses were performed. Robustness checks comprised (i) testing alternative operationalizations of predictors (e.g., categorical vs. continuous forms) to examine whether associations depended on variable coding; (ii) exploratory stepwise procedures (forward, backward, bidirectional) to compare data-driven selections with the prespecified model; (iii) exploratory testing of individual Barthel items and Braden scores to confirm their redundancy once the Barthel total was included; and (iv) additional checks of rare but clinically plausible predictors (e.g., antibiotic use, dementia, skin self-care) to assess stability despite small case numbers. Internal validation was conducted using bootstrap resampling (1,000 bias-corrected and accelerated samples).

#### Generalized estimating equations

To account for potential clustering at the nursing home level, a generalized estimating equations (GEE) model with a logit link, binomial distribution, and exchangeable correlation structure was fitted. Robust standard errors were applied, and all predictors from the final logistic regression were included.

All calculations were conducted using IBM SPSS Statistics, Version 30.

### Assessment of risk of bias and applicability

The risk of bias and concerns regarding applicability were evaluated using the Prediction Model Risk of Bias Assessment Tool (PROBAST)^42^. Although PROBAST was originally designed for systematic reviews, its application in this study aligns with guidance from the TRIPOD Cluster Explanation and Elaboration document^43^. When existing datasets are reused for prediction model development, structured assessments of bias are recommended. Because the dataset was not originally collected for prediction model development, PROBAST was applied to systematically examine potential sources of bias.

The PROBAST tool evaluates risk of bias across four domains (participants, predictors, outcome, analysis) and applicability across three domains (participants, predictors, outcome) using 20 signaling questions. Responses include “yes,” “probably yes,” “no,” “probably no,” or “no information.” Domains with only affirmative responses are rated as low risk, while any negative answer flags possible bias. “No information” reflects insufficient detail. Domain-level judgments are summarized as low, high, or unclear, with the overall risk classified as low only if all domains are rated low; a single high-risk domain results in an overall high-risk judgment. This structured assessment ensured transparency and facilitated consistent judgment of potential risks and applicability concerns.

## Results

### Participants and clusters

**Fig. 1:**
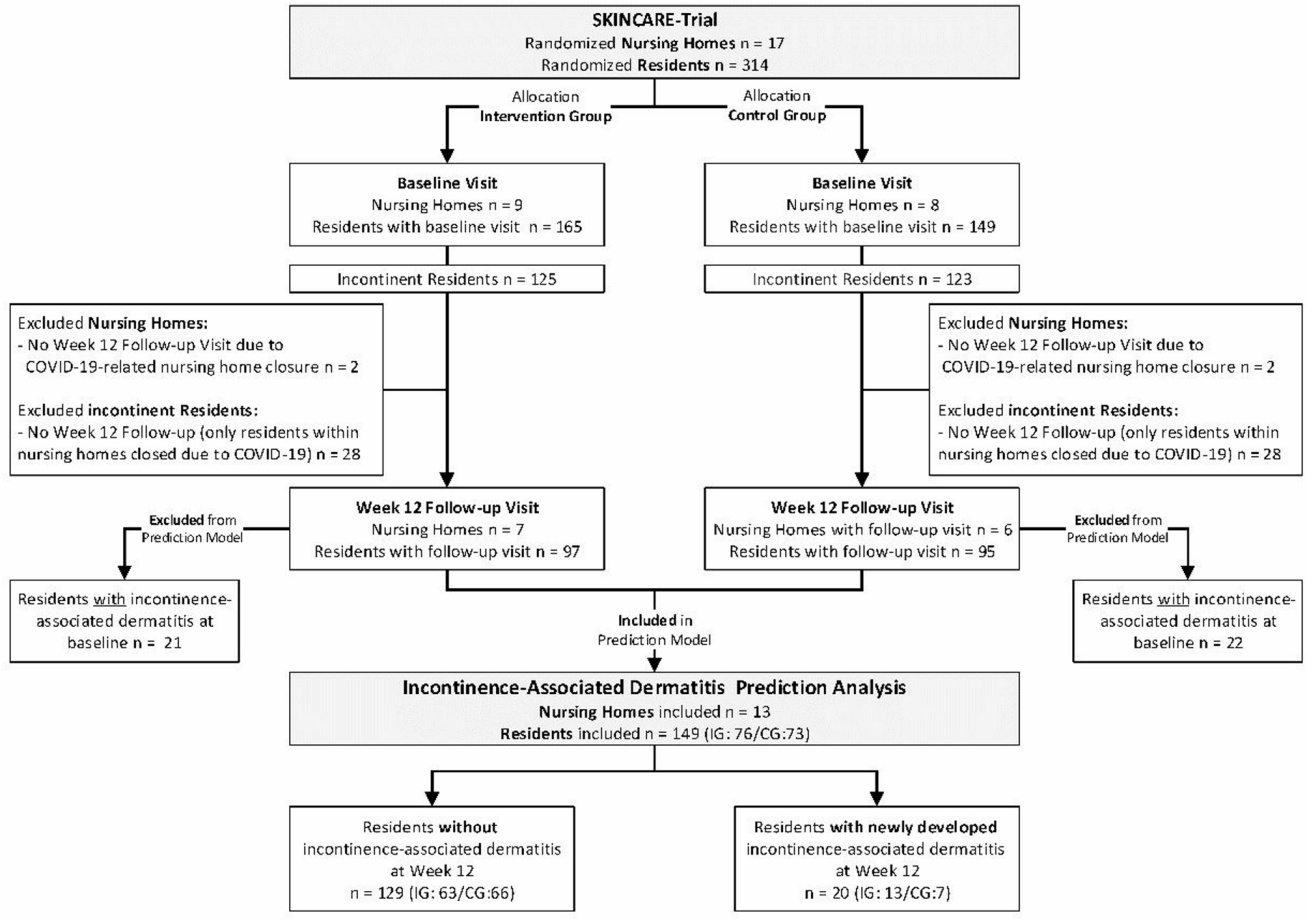
Flowchart of the data selection process for prediction analysis. The figure depicts the derivation of the analytic sample from the underlying SKINCARE cluster-randomized trial. Of 314 randomized residents across 17 nursing homes, only incontinent residents with both baseline and week-12 follow-up data were eligible. All incontinent residents without a week 12 follow-up originated from nursing homes that were temporarily closed due to COVID-19-related restrictions; no additional losses to follow-up occurred among residents from nursing homes that remained open. Participants with IAD at baseline were excluded. The final dataset included 149 incontinent residents from 13 nursing homes (intervention group, *n* = 76; control group, *n* = 73), among whom 20 developed new IAD during the 12-week study period. *IG* intervention group, *CG* control group.

At baseline, 314 residents from 17 nursing homes were enrolled in the underlying cluster-randomized controlled trial, of whom 248 (79%) were incontinent. Due to the COVID-19 pandemic, 4 clusters comprising 56 incontinent residents did not receive the 12-week follow-up visit. For the prediction analysis, only residents with data available at both baseline and week 12, documented urinary and/or fecal incontinence, and no signs of IAD at baseline were eligible. After excluding residents with IAD at baseline, the final analysis set comprised 149 residents from 13 clusters. Figure 1 provides a flow chart of the data selection process.

Preliminary analyses showed no statistically significant differences in IAD incidence between trial arms at either the individual or cluster level. Clustering by nursing home with respect to the outcome IAD was limited (ICC = 0.03), indicating minimal outcome clustering within facilities.

#### Baseline characteristics

Table 1 summarizes the baseline characteristics of the analyzed population, categorized by the presence or absence of incident IAD at week 12. The mean age was 84.4 (SD 7.8) years, and two thirds were female (67.1%). The mean BMI was 26.9 (SD 5.6) kg/m^2^. The mean Barthel Index total score was 38.5 (SD 22.8), indicating severe dependency in activities of daily living. One third of the residents (33.6%) had a GDS score of 4 to 7, reflecting moderate to very severe cognitive impairment. At baseline, all but one resident had urinary incontinence, while one resident was exclusively stool incontinent. Nearly half of the sample (47.0%, 70/149) had double incontinence. The mean length of stay was 35.9 (SD 34.8) months. At the week 12 follow-up visit, 20 of 149 residents presented with newly developed IAD, corresponding to a cumulative incidence of 13.4%. Most baseline characteristics were similar between groups, with differences mainly in those variables later identified as predictors.

**Table 1:** Baseline characteristics of analyzed residents and univariate group comparisons.

Variables	Newly developed IAD at week 12 *n* = 20	No IAD at week 12 *n* = 129		*p*-value
**Continuous Variables**			**MD (95% CI)**	
**Age** [years], mean (SD)	82.70 (6.56)	84.71 (7.95)	2.00 (−1.69–5.70)	0.227
**BMI** [kg/m^2^], mean (SD)	25.33 (5.43)	27.11 (5.65)	1.78 (−0.89–4.45)	0.190
**Residency duration** [months], mean (SD)	32.80 (27.23)	36.36 (35.95)	3.56 (−10.45–17.57)	0.608
**Braden Scale** [score], mean (SD)				
**Total Score**	14.85 (3.28)	16.26 (3.29)	1.41 (−0.16–2.97)	0.087
**Moisture Subscore**	2.75 (0.72)	3.05 (0.58)	0.30 (0.01–0.58)	**0.043**
**Friction Subscore**	1.80 (0.83)	2.19 (0.73)	0.39 (0.03–0.74)	**0.032**
**Barthel Index** [score], mean (SD)				
**Total Score**	25.50 (17.61)	40.54 (22.94)	15.04 (6.00–24.08)	**0.002**
**Dressing Subscore**	2.25 (3.02)	4.50 (3.05)	2.25 (0.80–3.69)	**0.003**
**Transfer Subscore**	5.75 (3.73)	8.99 (4.69)	3.24 (1.34–5.14)	**0.002**
**Controlling Bowels Subscore**	3.25 (4.38)	5.74 (4.76)	2.49 (0.25–4.73)	**0.030**
**TEWL** [g/m²/h]				
Arm	9.07 (2.11)	12.02 (8.99)	2.95 (1.12–4.77)	**0.002**
Leg	7.53 (2.76)	9.34 (5.38)	1.81 (0.24–3.38)	**0.025**
**Categorical Variables**			**OR (95% CI)**	
**Sex**, n (%)				
Female	11 (55.0)	89 (69.0)	**0.55 (0.21–1.43)**	0.215
Male	9 (45.0)	40 (31.0)		
**Underweight** (BMI < 18.5), n (%)	4 (20.0)	1 (0.8)	**32.00 (3.37–304.24)**	**< 0.001**
**Overweight** (BMI ≥ 25.0), n (%)	11 (55.0)	83 (64.3)	0.68 (0.26–1.76)	0.421
**Care Level**, n (%)				
II and III	8 (40.0)	84 (65.1)	0.36 (0.14–0.94)	**0.032**
IV and V	12 (60.0)	45 (34.9)	**2.80 (1.07–7.35)**	**0.032**
**GDS Stages**, n (%)				
GDS 1 to 3	10 (50.0)	89 (69.0)	0.45 (0.17–1.17)	0.094
GDS 4 to 7	10 (50.0)	40 (31.0)	**2.23 (0.86–5.77)**	0.094
**Incontinence Type**, n (%)				
Urinary	20 (100.0)	128 (99.2)	—	0.693
Fecal	15 (75.0)	56 (43.4)	3.91 (1.34–11.41)	**0.008**
Both	15 (75.0)	55 (42.6)	**4.04 (1.38–11.77)**	**0.007**
**Medication**, n (%)				
Influencing renin-angiotensin system	10 (50.0)	67 (51.9)	0.93 (0.36–2.37)	0.872
Corticosteroids	1 (5.0)	4 (3.1)	1.65 (0.17–15.51)	0.661
Diuretics	10 (50.0)	65 (50.4)	0.99 (0.38–2.53)	0.974
Sedatives	13 (65.0%)	77 (59.7%)	1.25 (0.47–3.36)	0.651
Antibiotics	2 (10.0%)	3 (2.3%)	**4.67 (0.73–29.86)**	0.076
Polypharmacy (≥ 5)	19 (95.0%)	114 (88.4%)	**2.50 (0.31–20.05)**	0.373
**Skin Conditions**, n (%)				
Xerosis cutis arms	16 (80.0%)	102 (79.1%)	1.06 (0.33–3.43)	0.924
Xerosis cutis legs	11 (55.0%)	113 (87.6%)	**0.17 (0.06–0.48)**	**< 0.001**
Skin tears	1 (5.0%)	12 (9.3%)	**0.51 (0.06–4.18)**	0.526
Pressure ulcers	3 (15.0%)	11 (8.5%)	**1.89 (0.48–7.48)**	0.356
Intertrigo	9 (45.0%)	46 (35.7%)	**1.48 (0.57–3.82)**	0.421
**Other Medical Conditions**, n (%)				
Dementia	7 (35.0%)	45 (34.9%)	1.01 (0.37–2.70)	0.992
Depression	3 (15.0%)	24 (18.6%)	0.77 (0.21–2.85)	0.697
Diabetes	7 (35.0%)	53 (41.1%)	0.77 (0.29–2.07)	0.606
Severe mobility impairment	7 (35.0%)	13 (10.1%)	**4.81 (1.63–14.19)**	**0.002**
**Other**, n (%)				
Pressure ulcer prevention device	14 (70.0%)	73 (56.6%)	**1.79 (0.65–4.95)**	0.258
Skin self-care ability	12 (60.0%)	53 (41.1%)	**2.15 (0.82–5.62)**	0.147

*BMI* Body Mass Index, *CI* confidence interval, *GDS* Global Deterioration Scale, *IAD* incontinence-associated dermatitis, *MD* mean difference, *OR* odds ratio, *SD* standard deviation, *TEWL* trans epidermal water loss.

Baseline characteristics of incontinent residents without IAD at baseline (*n* = 149; 20 with, 129 without new IAD at week 12). Incident IAD is defined as any newly developed IAD of category 1 A, 1B, 2 A or 2B at week 12 Bold values indicate statistical significance (*p* ≤ 0.05), odds ratios of ≤ 0.5 or ≥ 2, or mean differences considered relevant. Continuous variables are shown as mean (SD) and categorical variables as n (%). Group differences were analyzed using mean differences with 95% CIs and ORs.

#### Risk of bias assessment and applicability

Using the PROBAST tool, risk of bias was judged as low across all domains. The underlying trial applied clearly defined inclusion and exclusion criteria and enrolled a representative sample of nursing home residents aged 65 years and older. Predictors were uniformly assessed at baseline without knowledge of outcome data, and all variables included in the model are routinely available in clinical practice. The outcome, incident IAD at 12 weeks, was classified prospectively using the standardized GLOBIAD definition. None of the predictors were part of the outcome definition. The 12-week follow-up interval reflects an appropriate time interval between predictor assessment and outcome determination. Complete PROBAST assessment, including detailed judgement of each domain, is available in Appendix 1.

### Predictors

More than 50 baseline variables were initially examined as potential predictors of IAD development, covering demographics, functional and cognitive status, skin physiology parameters and skin conditions, comorbidities, and regular medication intake. Variables with neither statistical significance (*p* ≤ 0.05) nor prior plausibility based on the literature were excluded from further consideration. For constructs measured by total scores and subitems (e.g. Barthel Index, Braden Scale), both levels were screened to assess domain-specific information; they were not entered simultaneously in following multivariable modeling steps. The variables judged statistically, clinically, or theoretically relevant are displayed in Table 1.

#### Univariate group comparisons

In group comparisons, residents who developed IAD had substantially lower functional status than those who did not. This was evident for the Barthel Index total score (mean difference; MD 15.04, 95% CI 6.00–24.08, *p* = 0.002) and for subdomains including dressing (MD 2.25, 0.80–3.69, *p* = 0.003), transfer (MD 3.24, 1.34–5.14, *p* = 0.002), and bowel control (MD 2.49, 0.25–4.73, *p* = 0.030). Lower Braden subscores for moisture (MD 0.30, 0.01–0.58, *p* = 0.043) and friction (MD 0.39, 0.03–0.74, *p* = 0.032) also characterized incident cases. Regarding skin physiology, TEWL was lower among residents who developed IAD (arm: MD 2.95, 1.12–4.77, *p* = 0.002; leg: MD 1.81, 0.24–3.38, *p* = 0.025).

Among categorical variables, underweight residents (BMI < 18.5) were more frequent in the IAD group, as were those with high care dependency (Care Level IV–V), double incontinence, or severe mobility impairment. Visible xerosis on the legs was less common among residents who developed IAD. Differences for other variables, such as antibiotic use or skin self-care ability, did not reach statistical significance but were retained for further examination based on clinical plausibility and literature.

#### Univariate logistic regression

In univariate logistic regression analyses, several predictors showed strong associations with incident IAD (Table 2). Being underweight (BMI < 18.5) was the strongest risk factor (OR 32.00, 95% CI 3.14–326.87, *p* = 0.004). Residents with double incontinence had more than a fourfold increased risk (OR 4.04, 95% CI 1.31–12.70, *p* = 0.012), and severe mobility impairment was likewise strongly associated (OR 4.81, 95% CI 1.58–14.78, *p* = 0.003). In contrast, visible signs of xerosis on the legs were associated with lower risk (OR 0.17, 95% CI 0.05–0.47, *p* < 0.001).

**Table 2:** Univariate logistic regression analyses for candidate predictors.

Variables		OR (95% CI)	*p*-value
**Continuous Variables**			
**Age** [years]		0.97 (0.91–1.03)	0.185
**BMI** [kg/m^2^]		0.94 (0.85–1.03)	0.194
**Duration of residency** [months]		1.00 (0.98–1.01)	0.637
**Braden Scale** [score]			
**Total Score**		0.88 (0.76–1.02)	0.076
**Moisture Subscore**		**0.46 (0.22–0.99)**	**0.040**
**Friction Subscore**		**0.50 (0.26–0.96)**	**0.047**
**Barthel Index** [score]			
**Total Score**		0.97 (0.95–0.99)	**0.003**
**Dressing Subscore**		0.77 (0.65–0.92)	**0.006**
**Transfer Subscore**		0.85 (0.76–0.95)	**< 0.001**
**Controlling Bowels Subscore**		0.89 (0.80–0.99)	**0.021**
**TEWL** [g/m²/h]			
Arm		0.90 (0.77–1.04)	0.162
Leg		0.86 (0.71–1.06)	0.158
**Categorical Variables**	**Reference category**		
**Female Sex**	male sex	**0.55 (0.19–1.46)**	0.238
**Underweight** (BMI < 18.5)	absence	**32.00 (3.14–326.87)**	**0.004**
**Overweight** (BMI ≥ 25.0)	absence	0.68 (0.25–1.81)	0.437
**Care Level IV or V**	Care level II/III	**2.80 (1.02–7.86)**	**0.034**
**GDS 4 to 7**	GDS 1 to 3	**2.23 (0.83–5.91)**	0.101
**Incontinence Double** Urinary *and* Fecal	Incontinence single	**4.04 (1.31–12.70)**	**0.012**
**Medication**			
Influencing renin-angiotensin system	absence	0.93 (0.35–2.39)	0.871
Corticosteroids	absence	1.65 (0.15–15.67)	0.674
Diuretics	absence	0.99 (0.37–2.53)	0.967
Sedatives	absence	1.25 (0.47–3.36)	0.659
Antibiotics	absence	**4.67 (0.69–31.24)**	0.106
Polypharmacy (≥ 5)	absence	**2.5 (0.28–21.63)**	0.378
**Skin conditions**			
Xerosis cutis arms	absence	1.06 (0.32–3.48)	0.921
Xerosis cutis legs	absence	**0.17 (0.05–0.47)**	**< 0.001**
Skin tears	absence	**0.51 (0.06–4.09)**	0.528
Pressure ulcers	absence	1.89 (0.45–7.34)	0.352
Intertrigo	absence	1.48 (0.55–3.94)	0.419
**Other Medical Conditions**			
Dementia	absence	1.01 (0.36–2.74)	0.985
Depression	absence	0.77 (0.19–2.76)	0.642
Diabetes	absence	0.77 (0.27–2.03)	0.606
Severe mobility impairment	absence	**4.81 (1.58–14.78)**	**0.003**
Other			
Pressure ulcer prevention device	absence	1.79 (0.61–4.82)	0.254
Skin self-care ability	absence	**2.15 (0.79–5.58)**	0.129

*BMI* Body Mass Index, *CI* confidence interval, *GDS* Global Deterioration Scale, *IAD* incontinence-associated dermatitis, *MD* mean difference, *OR* odds ratio, *SD* standard deviation, *TEWL* trans epidermal water loss.

Continuous predictors are presented per unit increase, categorical predictors relative to the indicated reference category. ORs with bias-corrected and accelerated 95% CIs were obtained from 1000 bootstrap samples.

Functional dependency also showed consistent effects across domains: each additional point on the Barthel Index reduced IAD odds by 3% (OR 0.97, 95% CI 0.95–0.99, *p* = 0.003). Lower Braden subscore for moisture (OR 0.46, 95% CI 0.22–0.99, *p* = 0.040) and friction (OR 0.50, 95% CI 0.26–0.96, *p* = 0.047) were also associated with higher risk.

Variables that did not reach statistical significance but were considered conceptually relevant, such as antibiotic use (OR 4.67, 95% CI 0.69–31.24, *p* = 0.106), skin self-care ability (OR 2.15, 95% CI 0.79–5.58, *p* = 0.129), and dementia (OR 1.01, 95% CI 0.36–2.74, *p* = 0.985), were retained for exploratory testing in subsequent multivariable modeling.

#### Collinearity analysis

Very high correlations were observed within the Braden and Barthel instruments, for example between the Braden total score and its Mobility subscore (*r* = 0.91), the Barthel total score and its subdomains Standing/Mobility (*r* = 0.85) and Transfer (*r* = 0.79), and between the Barthel and Braden Mobility scores (*r* = 0.81). Strong associations were also present among indicators of functional dependency such as care level, GDS, and double incontinence (phi coefficients up to 0.61). To avoid collinearity, such variables were not entered simultaneously in the same model. Instead, either total scores or selected subdomains were retained depending on statistical performance and conceptual relevance. No problematic collinearity was detected among the skin physiology variables, BMI, or age. These findings guided the selection of a reduced predictor set for subsequent multivariable modeling.

### Prediction model development

#### Multivariable logistic regression

Variables retained after univariate screening and collinearity assessment were entered simultaneously into a multivariable logistic regression model. Among highly correlated measures, only one variable per construct was retained. The Barthel Index was preferred over the Braden Scale and its sub-scores because of stronger and more consistent associations with IAD and to avoid redundancy (*r* > 0.8). Individual Barthel items were tested but did not improve prediction beyond the total score. For body composition, the dichotomous underweight category (BMI < 18.5 kg/m²) showed the strongest univariable effect but was excluded from the final model due to very small subgroup size and unstable odds ratios. Continuous BMI was retained, while underweight was considered only in sensitivity analyses.

In an exploratory analysis, potential interactions between continence type and functional ability were examined. This approach was guided by clinical reasoning and supported by the observed univariate association of double incontinence with IAD risk. The analysis revealed an interaction, indicating that the association between higher Barthel Index scores and lower IAD risk differed by continence status. Specifically, the protective effect of higher functional independence was weakened among residents with double incontinence. Consequently, the interaction term between double incontinence and Barthel Index total score was included in the final model.

The final model comprised continuous BMI, Barthel Index total score, visible signs of xerosis on the legs, severe mobility impairment, and the interaction between double incontinence and Barthel Index total score. Based on 20 outcome events, the final model included five estimated parameters, corresponding to an events-per-parameter ratio of 4.0. Alternative model specifications examined during sensitivity analyses ranged from three to six estimated parameters, resulting in events-per-parameter ratios between 3.3 and 6.7.

Exploratory stepwise procedures (forward, backward, bidirectional) supported this specification. The model combined statistical performance with clinical interpretability, ensuring that predictors were both stable and practically relevant. Variance inflation factors (VIF) ranged from 1.0 to 2.9, confirming the absence of problematic multicollinearity.

#### GEE analysis

To verify that predictive effects were not driven by clustering at the nursing home level, the final model was refitted using GEE with an exchangeable correlation structure. Results were consistent with the standard logistic regression: Barthel Index, xerosis of the legs, severe mobility impairment, and the interaction term remained highly significant predictors, while BMI showed the same negative but non-significant association. This suggests that the predictive effects were not driven by nursing home level clustering. Results of the final cluster-adjusted model are summarized in Table 3.

**Table 3:** Final cluster-adjusted multivariable logistic regression model.

Predictor	B (SE)	OR (95% CI)	Wald χ²	*p*-value
**BMI** [per 1 kg/m^2^]	−0.068 (0.043)	0.93 (0.86–1.02)	2.48	0.115
**Barthel Index Total Score ** [per 1 point]	−0.120 (0.028)	0.89 (0.84–0.94)	18.17	< 0.001
**Xerosis Legs** [present]	−1.696 (0.456)	0.18 (0.07–0.45)	13.86	< 0.001
**Severe mobility impairment** [present]	1.488 (0.440)	4.43 (1.87–10.47)	11.46	< 0.001
Interaction: **Double incontinence ×** **Barthel Index** ^*^	0.137 (0.031)	1.15 (1.08–1.22)	19.59	< 0.001

*GEE* generalized estimating equations, *B* regression coefficient, *SE* standard error, *Wald χ²* Wald chi-square statistic, *OR* odds ratio, *CI* confidence interval, *BMI* Body Mass Index, *IAD* incontinence-associated dermatitis.

ORs are obtained as exp(B), representing the multiplicative change in the odds of developing IAD per one-unit increase (continuous variables) or presence versus absence (binary variables). Estimates are based on GEE with logit link, binomial distribution, exchangeable correlation structure, and robust standard errors.

^*^The interaction term (Double incontinence × Barthel Index) indicates that the protective effect of functional independence (higher Barthel scores) is reduced in residents with double incontinence.

#### Sensitivity analysis

Several sensitivity analyses confirmed the robustness of the final model. Reintroducing the underweight category (BMI < 18.5 kg/m²) yielded consistent risk directions but unstable estimates due to the very small subgroup size. Alternative operationalizations, including continuous versus categorical BMI, Barthel total versus item scores, and Braden total versus subscale scores, produced comparable results. Including trial group assignment (intervention vs. control) did not affect effect directions or magnitudes, indicating no contamination bias. Exploratory stepwise procedures (forward, backward, bidirectional) consistently retained the core predictors (BMI, Barthel Index, xerosis of the legs, and severe mobility impairment). Rare predictors such as antibiotic use, dementia, and skin self-care ability showed no reproducible effects. Internal validation using 1,000 bias-corrected and accelerated bootstrap samples confirmed the stability of all coefficients, including the interaction term (double incontinence × Barthel Index) which remained robust across all sensitivity checks.

#### Model performance

**Fig. 2:**
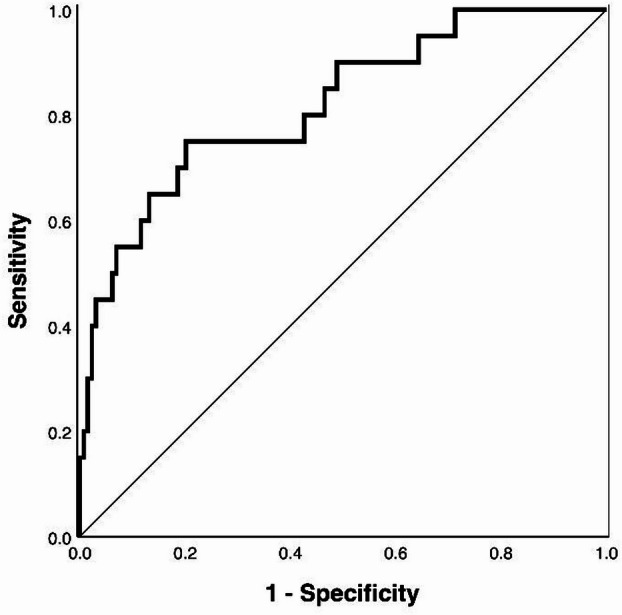
Receiver Operating Characteristic (ROC) curve of the final prediction model. The area under the curve (AUC) is shown with 95% confidence interval. Diagonal line represents the reference line (AUC = 0.5).

Discrimination of the final multivariable model was good, with an area under the ROC curve (AUC) of 0.819 (95% CI: 0.715–0.923) (Fig. 2). The Hosmer–Lemeshow goodness-of-fit test was non-significant (χ² = 5.80, df = 8, *p* = 0.670), and the mean Brier score was 0.086, both indicating good calibration and overall predictive accuracy.

Classification performance varied across probability thresholds Table 4. Specificity reached 100% at a cutoff of 0.70 but sensitivity declined to 10%. At a cutoff of 0.10, sensitivity was 75% and specificity 64%. A grid search identified an optimal threshold of approximately 0.14, balancing sensitivity (75.0%) and specificity (76.7%) with a balanced accuracy of 75.9%.

**Table 4:** Receiver operating characteristic (ROC) analysis of the final cluster-adjusted prediction model at different probability thresholds.

Cutoff Probability	Sensitivity [%]	Specificity [%]	Balanced Accuracy [%]
0.1	75.0	64.3	69.6
0.3	50.0	93.0	71.5
0.5	30.0	97.7	63.9
0.7	10.0	100	55.0
≈ 0.14*	75.0	76.7	75.9

*ROC* Receiver Operating Characteristic, *AUC* area under the curve, *IAD* incontinence-associated dermatitis.

Model performance is shown for several cutoff probabilities with corresponding sensitivity, specificity, and balanced accuracy. Sensitivity represents the proportion of true positives correctly classified, specificity the proportion of true negatives correctly classified, and balanced accuracy the mean of both.

*The optimal cutoff (≈ 0.14) was identified via grid search, providing nearly equal sensitivity and specificity.

**Fig. 3:**
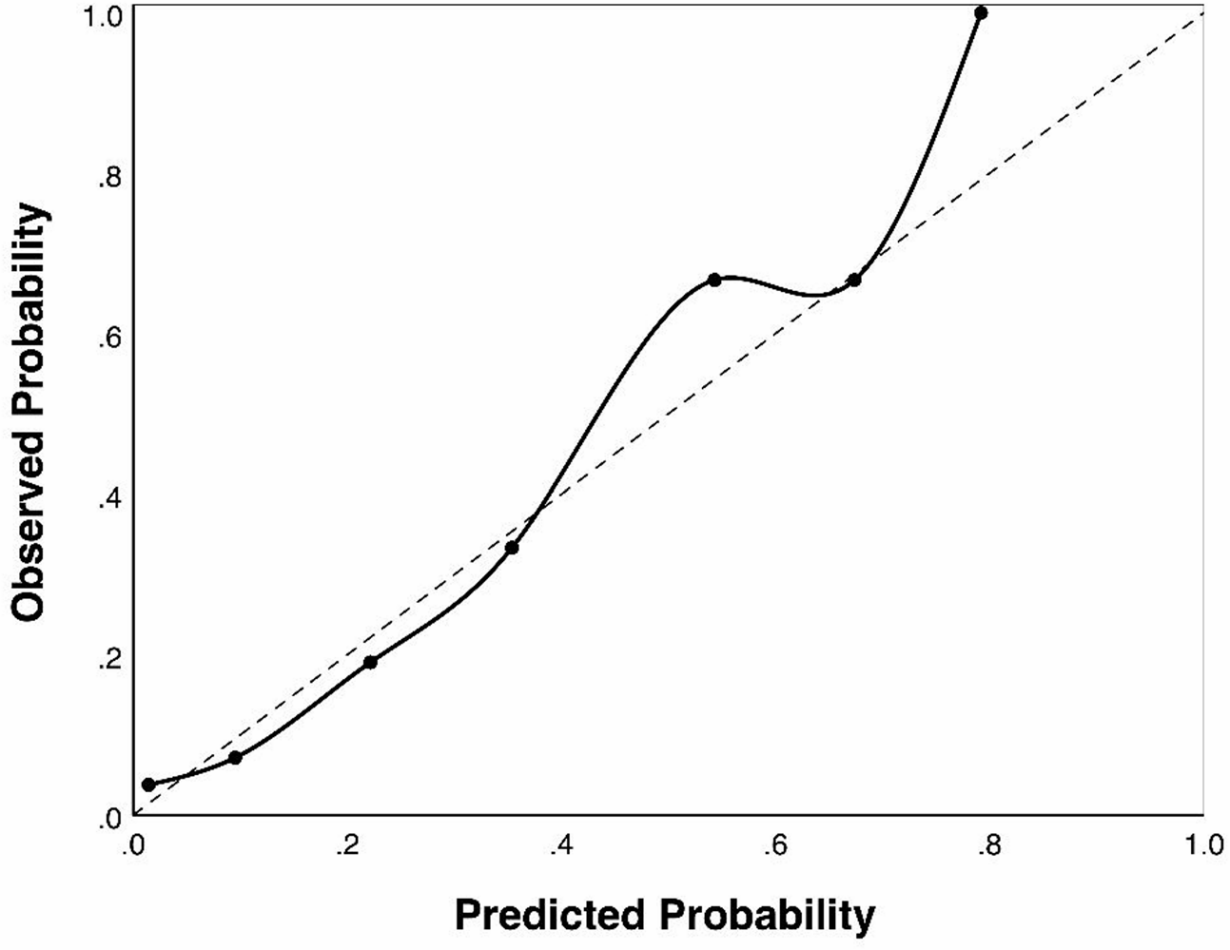
Calibration plot of the final prediction model. The observed incidence is plotted against the predicted probabilities. The dashed line represents perfect calibration (prediction equals observation), and the solid line represents model-predicted values.

Calibration analysis showed a slope of 1.0 (SE = 0.24, Wald χ² = 17.63, *p* < 0.001) and calibration-in-the-large (average difference between predicted and observed risk) close to zero. The calibration plot (Fig. 3) indicated close alignment with the ideal diagonal at lower and intermediate predicted risks ranges, with modest overprediction at higher probabilities.

## Discussion

This study aimed to identify individual risk factors for the development of IAD and to develop a prognostic model for incontinent long-term care residents aged 65 years and older. Using data from a cluster-randomized controlled trial, the final, cluster-adjusted multivariable model identified four key predictors: lower functional status (Barthel Index), severe mobility impairment, absence of visible xerosis on the lower extremities, and an interaction effect between double incontinence and functional dependency.

### Individual risk factors

Several baseline characteristics showed associations with incident IAD in the univariate analyses. The strongest effects were observed for double incontinence and severe mobility impairment, each increasing the odds of IAD more than fourfold. Residents with lower Barthel Index scores were also more likely to develop IAD, reflecting that dependency in daily activities predisposes to prolonged skin exposure and reduced self-care. Underweight residents (BMI < 18.5 kg/m²) showed markedly elevated odds of IAD in univariate analyses. However, this result was based on few cases and should be interpreted cautiously, as the very small subgroup size resulted in sparse data and unstable estimates, precluding inclusion in the final multivariable model. Residents showing visible signs of xerosis on the legs were less likely to develop IAD. These univariate patterns mirror the mechanisms described in previous research, where mobility restrictions, double incontinence, and nutritional factors consistently emerge as important risks for IAD^21,22,44^. They provided the conceptual basis for the multivariable analysis, which aimed to determine which of these factors remained independently relevant once potential overlap and confounding were controlled for.

### Predictors in the final model

In the cluster-adjusted multivariable model, four predictors remained significant: functional dependency as measured by the Barthel Index, severe mobility impairment, visible xerosis of the legs, and the interaction between double incontinence and functional dependency. Continuous BMI was also retained as a covariate, although its effect did not reach statistical significance.

Lower Barthel Index scores were associated with increased IAD risk, consistent with previous evidence identifying limited self-care and mobility restrictions as key determinants of IAD in older adults^21,44^.

Severe mobility impairment remained one of the strongest independent risk factors, confirming the results of the univariate analyses. This aligns with systematic review findings providing moderate-quality evidence for mobility limitations as a prognostic factor^21^and with international expert consensus ranking immobility among the most critical risks for IAD development^22^. Importantly, this variable captures a dimension of severely restricted mobility often related to underlying neurological conditions, extending beyond the general functional dependency measured by the Barthel Index Mobility item. The absence of collinearity between these variables supports their distinct yet complementary contributions to IAD risk.

Interestingly, visible signs of xerosis on the legs were associated with a lower likelihood of developing IAD. Xerosis cutis is characterized by reduced hydration of the stratum corneum^45^. A possible explanation is that increased exposure to moisture due to incontinence may compensate for the underlying skin dryness rather than lead to overhydration.

A similar pattern has been described in pediatric populations, where atopic dermatitis, a condition characterized by impaired epidermal barrier function and commonly accompanied by xerosis, typically spares the diaper area. Despite the continuous moisture and occlusion in this region, the skin often appears intact rather than showing eczematous or irritative changes, which has been interpreted as a local protective effect. Although this phenomenon has been described specifically in pediatric populations, it may nonetheless reflect broader mechanisms by which microclimate and moisture exposure influence skin integrity, offering a possible explanation for the lower IAD risk observed in residents with drier skin phenotypes^46^.

In contrast to previous studies that often report a single global dryness rating for the entire body surface, this study employed region-specific xerosis assessments, capturing dryness of distinct anatomical sites. This approach likely reduced misclassification, as skin dryness and microclimate can vary substantially across body regions^45^. Thus, xerosis of the legs may represent a marker of a generally drier skin phenotype. Alternatively, visible xerosis may have triggered preventive care routines, such as emollient application or gentler cleansing, indirectly reducing the likelihood of skin breakdown^47^. Its stable inverse association in the final model suggests that interindividual differences in skin microclimate and moisture regulation may be clinically relevant and warrant consideration in future IAD risk stratification and prevention research.

The interaction between double incontinence and functional dependency may indicate a more complex risk pattern. The expected protective effect of higher functional independence appeared to diminish among residents with double incontinence. This suggests that, while continence status and functional ability are each important in their own right, their combination captures an additional aspect of vulnerability that is not evident when the two are considered separately. Although the underlying mechanisms remain uncertain, this finding identifies a potential area for further investigation and may help refine understanding of how functional capacity and moisture exposure jointly contribute to IAD risk, underscoring the need to assess both domains together in clinical evaluation and prevention.

Overall, the final model reflects a coherent and clinically meaningful risk profile in which reduced functional capacity and mobility, compounded by double incontinence, markedly increase vulnerability to IAD, while xerosis of the legs may indicate a comparatively less IAD-prone skin type. Although BMI did not reach statistical significance, its consistent direction of effect supports the notion that lower body mass and potential malnutrition further contribute to skin fragility. Together, these predictors represent a multidimensional vulnerability framework encompassing functional, mobility-related, and skin-physiological components central to IAD pathogenesis.

### Model performance

The final model demonstrated good discrimination and calibration, indicating adequate fit and predictive precision within the available sample. The AUC of 0.819 reflects solid ability to distinguish between residents with and without incident IAD. Model calibration metrics (Hosmer–Lemeshow test, Brier score, calibration slope) also indicated good fit, although precision is limited by the modest number of events. While these indicators are promising, external validation remains necessary to confirm model performance.

### Sensitivity analyses

Sensitivity analyses confirmed the robustness of the model across variable coding and modelling strategies. The interaction between double incontinence and functional dependency remained stable across checks, suggesting the signal is not driven by modelling choices. Also, the inclusion of trial group assignment (intervention vs. control) did not alter estimates. These results support the internal validity and clinical plausibility of the identified predictors.

### Differences from previous prognostic IAD research

Previous prognostic research on incontinence associated dermatitis has primarily focused on individual risk factors rather than comprehensive multivariable models, and few studies have considered interdependencies among predictors. Most existing analyses were conducted in acute or intensive care settings, where short observation periods and distinct patient profiles limit transferability to long-term care^21^. The present model confirms key findings from prior work, like the central role of immobility or double incontinence, but extends this evidence base by quantifying their combined influence within a representative long-term care population. The observed interaction between double incontinence and functional dependency indicates that the apparent protective effect of functional independence may be attenuated when residents are simultaneously exposed to urine and feces, a relationship not previously quantified in prognostic IAD research.

This model also introduces new perspectives by integrating functional, mobility-related, and skin physiological factors within a single predictive framework. Unlike earlier studies that linked xerosis to increased vulnerability, the present analysis revealed an inverse association for dryness of the legs, which cannot be conclusively explained within the current dataset. The use of region-specific skin assessments in the underlying clinical trial may partly account for this difference, as previous research often relied on global dryness ratings that obscure regional variability. By focusing on a representative long-term care population rather than short-term hospital cohorts, this model also captures chronic exposure patterns and complex comorbidities that better reflect the geriatric context of IAD risk. Collectively, these features distinguish the present model as a more comprehensive and context-specific contribution to prognostic IAD research.

### Implications for clinical practice

The final model comprises variables that are routinely assessed in long-term care, which supports its potential applicability in daily practice. Functional dependency, mobility status, continence type, and body weight are standard components of geriatric nursing assessments and could therefore be readily incorporated into structured skin care risk evaluations. The results emphasize the importance of addressing exposure-related and functional factors simultaneously: even residents who appear relatively independent may remain at high risk when double incontinence is present, whereas immobile or functionally dependent residents require intensified preventive care regardless of continence type.

Systematic skin inspection also emerges as a key implication. Incorporating such observations into routine skin assessments could help individualize prevention strategies. In addition, evidence from previous studies suggests that both undernutrition and overweight can compromise skin integrity, reinforcing the importance of nutritional monitoring as a modifiable preventive factor.

Overall, these findings indicate that better use of information already available in routine care may be more effective than introducing new instruments. Strengthening caregivers’ systematic attention to existing assessment data and interpreting it consistently could substantially improve early risk recognition. Embedding such risk profiles into care planning can guide timely preventive actions, such as optimizing continence care, maintaining mobility, supporting adequate nutrition, and adapting skin cleansing and protection routines. In this way, the model provides a practical contribution for translating evidence-based risk assessment into individualized preventive strategies in long-term care settings.

At the same time, the present model represents an initial development and internal validation step. Although it identifies clinically meaningful risk patterns using routinely available information, further work is required before it can be translated into a standardized, user-oriented clinical tool. In particular, external validation in independent populations is necessary prior to the development and implementation of simplified risk scores or decision support formats.

### Limitations

Several limitations should be considered when interpreting these findings. This was a secondary analysis of a cluster randomized controlled trial that was not originally powered for prognostic modeling. The limited number of outcome events increases the risk of overfitting and reduced precision, although model reduction and bootstrapping were applied to enhance stability.

In addition, the prediction horizon of 12 weeks was defined by the follow-up structure of the underlying trial, with outcome assessments conducted at baseline, week 12, and week 24. This data structure did not allow modeling alternative or more granular time horizons, nor time-to-event analyses that might more precisely capture the temporal dynamics of IAD onset.

Residents from nursing homes that were temporarily closed due to COVID-19-related restrictions had no week 12 outcome assessment and were therefore not included in the prediction analysis. This resulted in missing outcome data at week 12 due to cluster-level loss to follow-up, which may have introduced selection bias. However, no additional individual-level drop-outs occurred, and missingness was unrelated to resident characteristics or outcome status.

Some potentially relevant variables, such as stool frequency or consistency, could not be included due to incomplete or proxy reported data, which may have introduced measurement bias. Residual confounding by unmeasured factors, including hygiene routines or product use, cannot be excluded. While the sample was representative of the local long-term care population, generalizability to other settings or healthcare systems remains uncertain. Finally, the model has not yet undergone external validation and should therefore be regarded as hypothesis generating until confirmed in independent populations, ideally using larger and prospective studies.

## Conclusion

This study identified a multidimensional profile of predictors for the development of IAD in incontinent long-term care residents aged 65 years and older. The final, cluster-adjusted prognostic model highlighted the combined relevance of functional dependency, severe mobility impairment, double incontinence, and skin condition. These factors together define a vulnerability profile in which physical dependency, mobility restriction, and moisture exposure interact in the context of IAD development.

The model demonstrated good discriminatory performance and satisfactory calibration, suggesting that routinely available clinical data can support reliable risk stratification in this population. These findings provide a foundation for developing simple, evidence-based assessment tools and for integrating risk based preventive strategies into routine nursing care.

## Supplementary Information

Below is the link to the electronic supplementary material.

Supplementary Material 1

## Data Availability

The data that support the findings of this study are available from the corresponding author upon reasonable request.
