# Supplementary material for: Identification of risk factors and development of a multivariable prognostic prediction model for incontinence-associated dermatitis in older nursing home residents
Source: Sci Rep. 2026 Feb 18;16:7163. doi: 10.1038/s41598-026-40416-7 (PMC12920631; doi:10.1038/s41598-026-40416-7)
Supplement: Supplementary file 1 — Supplementary Material 1 [file 41598_2026_40416_MOESM1_ESM.pdf]

Appendix 1: PROBAST-Assessment

DOMAIN 1: Participants			
**A. Risk of Bias**			
Describe the sources of data and criteria for participant selection:<br>Data from a cluster-randomized controlled trial conducted in Berlin, Germany, were used for developing the prediction model. The inclusion and exclusion criteria were appropriately defined to ensure that participants were representative of the population of interest (nursing home residents aged 65 years or older without incontinence-associated dermatitis at baseline). This approach corresponds well to an unselected population of interest, as participants were not pre-selected based on specific conditions beyond age and the absence of incontinence-associated dermatitis at baseline.			
		Dev	Val
1.1 Were appropriate data sources used, e.g. cohort, RCT or nested case-control study data?		Yes	n.a.
1.2 Were all inclusions and exclusions of participants appropriate?		Yes	n.a.
**Risk of bias introduced by selection of participants**	**RISK:**<br>*(low/ high/ unclear)*	**Low**	n.a.
Rationale of bias rating:<br>The risk of bias for this domain is judged to be low. An appropriate data source, a cluster-RCT (1.1: Yes), was used, aligning well with the study's objective of developing a prediction model for incontinence-associated dermatitis risk. All inclusions and exclusions were appropriate (1.2: Yes), with clear criteria ensuring that participants with incontinence-associated dermatitis at baseline were excluded. This aligned the study population with the intended focus on predicting development of incontinence-associated dermatitis. The data were collected directly by the same study team, which ensured consistency and systematic methodology in participant assessment. Additionally, the study population is a representative sample of nursing home residents aged 65 and older with minimal restrictions, enhancing the relevance of findings to this population.			

**DOMAIN 1: Participants**			
**B. Applicability**			
Describe included participants, setting and dates:<br>The study included long-term care nursing home residents aged 65 years or older, who were substantially care-dependent and resided in one of the participating facilities. The study was conducted over a period of three years across multiple locations in Berlin, Germany, from April 2019 to June 2021.			
**Concern that the included participants and setting do not match the review question**	**CONCERN:**<br>*(low/ high/ unclear)*	**Low**	n.a.
Rationale of applicability rating:<br>The applicability concern for this domain is judged to be low. The study population, consisting of nursing home residents aged 65 and older, closely matches the target population for the prediction model. The minimal inclusion criteria enhance the generalizability of the model to a broad range of nursing home residents, making the findings highly applicable to similar care settings.			

**DOMAIN 2: Predictors**			
**A. Risk of Bias**			
List and describe predictors included in the final model, e.g. definition and timing of assessment:<br>The predictors in the final model (Barthel Index total score, Body Mass Index, severe mobility impairment, xerosis on the legs, and double incontinence) were consistently defined and uniformly assessed across all participants at baseline and week 12 by the same study team.			
		Dev	Val
2.1 Were predictors defined and assessed in a similar way for all participants?		Yes	n.a.
2.2 Were predictor assessments made without knowledge of outcome data?		Yes	n.a.
2.3 Are all predictors available at the time the model is intended to be used?		Yes	n.a.
**Risk of bias introduced by predictors or their assessment**	**RISK:**<br>*(low/ high/ unclear)*	**Low**	n.a.

<table>
<tr><td colspan="4">DOMAIN 2: Predictors</td></tr>
<tr><td colspan="4">Rationale of bias rating:
The risk of bias for this domain is judged to be low. All predictors were consistently defined and assessed in the same manner for all participants (2.1: Yes), with no variation in data collection methods across individuals. Predictor assessments were made without knowledge of the outcome data (2.2: Yes), as all data were collected at baseline and prior to the outcome assessment, ensuring that assessments were unbiased. Additionally, all predictors are readily available and practical to use in clinical settings, making them accessible at the time the model is intended to be applied (2.3: Yes). These factors collectively support a low risk of bias in this domain and reinforce the model’s applicability in real-world settings.</td></tr>
<tr><td colspan="4">B. Applicability</td></tr>
<tr><td>Concern that the definition, assessment or timing of predictors in the model do not match the review question</td><td>CONCERN:
(low/ high/ unclear)</td><td>Low</td><td>n.a.</td></tr>
<tr><td colspan="4">Rationale of applicability rating:
The applicability concern for this domain is judged to be low. The selected predictors are routinely collected in standard nursing home environments, ensuring that their use aligns well with typical clinical practice. The model's reliance on uniform definitions, including specific clinical scores and ICD-11 codes, supports consistency and standardization in predictor assessment across settings. This minimizes variability in predictor measurement and ensures the model's applicability across different clinical contexts. Additionally, no specialized or advanced techniques were required to measure these predictors, further reducing any applicability concerns for this domain.</td></tr>
</table>

DOMAIN 3: Outcome			
**A. Risk of Bias**			
Describe the outcome, how it was defined and determined, and the time interval between predictor assessment and outcome determination:<br>The outcome was defined according to Global Incontinence-Associated Dermatitis Categorisation Tool (GLOBIAD), and incontinence-associated dermatitis was coded using ICD-11 by experienced study physicians who were familiar with both the population and the setting. All assessments were conducted by the same study team across all participants and clusters. Predictor information was collected at baseline, and the determination of incontinence-associated dermatitis was conducted at a follow-up interval of 12 weeks.			
		Dev	Val
3.1 Was the outcome determined appropriately?		Yes	n.a.
3.2 Was a pre-specified or standard outcome definition used?		Yes	n.a.
3.3 Were predictors excluded from the outcome definition?		Yes	n.a.
3.4 Was the outcome defined and determined in a similar way for all participants?		Yes	n.a.
3.5 Was the outcome determined without knowledge of predictor information?		Yes	n.a.
3.6 Was the time interval between predictor assessment and outcome determination appropriate?		Yes	n.a.
**Risk of bias introduced by the outcome or its determination**	**RISK:**<br>*(low/ high/ unclear)*	**Low**	n.a.
Rationale of bias rating:<br>The risk of bias for this domain is judged to be low. The outcome (development of incontinence-associated dermatitis) was determined using the standardized GLOBIAD definition and coded with ICD-11, which are pre-specified and widely accepted criteria (3.2: Yes), ensuring consistent and objective outcome determination (3.1: Yes). Assessments were			

**DOMAIN 3: Outcome**			
carried out exclusively by trained study physicians experienced in this population, with the same team performing all assessments across clusters, further reducing variability and ensuring uniformity in outcome definition and determination for all participants (3.4: Yes). None of the predictors in the model were used in defining incontinence-associated dermatitis, avoiding incorporation bias (3.3: Yes). Additionally, the assessors were blinded to predictor information, as the prediction model was developed after data collection, ensuring that outcome determination was not influenced by predictor knowledge (3.5: Yes). The 12-week follow-up period between baseline predictor collection and outcome determination was appropriate for capturing new occurrences of incontinence-associated dermatitis (3.6: Yes).			
**B. Applicability**			
At what time point was the outcome determined:<br>The outcome, development of incontinence-associated dermatitis, was determined at a 12-week follow-up after baseline predictor assessment.<br>*If a composite outcome was used, describe the relative frequency/distribution of each contributing outcome:*<br>n.a.			
**Concern that the outcome, its definition, timing or determination do not match the review question**	**CONCERN:**<br>*(low/ high/ unclear)*	**Low**	n.a.
Rationale of applicability rating:<br>The applicability concern for this domain is judged to be low. The use of the standardized GLOBIAD definition and ICD-11 coding ensures that the outcome aligns with clinical standards, enhancing relevance to practice. Additionally, the consistent timing of outcome assessment at 12 weeks supports the model's applicability to similar clinical settings, where periodic follow-up is standard.			

**DOMAIN 4: Analysis**
**Risk of Bias**
Describe numbers of participants, number of candidate predictors, outcome events and events per candidate predictor:<br><br>The analysis included n = 149 participants with 5 predictors. A total of n = 20 incontinence-associated dermatitis events were observed, resulting in an events per variable (EPV) ratio of less than 10. This may affect the model's stability and increase the risk of overfitting.
Describe how the model was developed (for example in regards to modelling technique (e.g. survival or logistic modelling), predictor selection, and risk group definition):<br><br>The model was developed using logistic regression and Generalized Estimating Equations (GEE) to account for data clustering within nursing homes. Predictor selection for the multivariable model was based on clinical relevance and literature review, rather than univariable analysis alone.
Describe whether and how the model was validated, either internally (e.g. bootstrapping, cross validation, random split sample) or externally (e.g. temporal validation, geographical validation, different setting, different type of participants):<br><br>Internal validation was conducted using bootstrapping with 1,000 bootstrap samples to assess the robustness of the model and adjust for potential optimism.
Describe the performance measures of the model, e.g. (re)calibration, discrimination, (re)classification, net benefit, and whether they were adjusted for optimism:<br><br>Model performance was evaluated in terms of calibration and discrimination. Discriminatory ability was assessed using the Area Under the Curve (AUC), while calibration was assessed with calibration plots and the Hosmer-Lemeshow test.
Describe any participants who were excluded from the analysis:<br><br>All participants eligible for this analysis were included; no cases were excluded because of missing data.
Describe missing data on predictors and outcomes as well as methods used for missing data:<br><br>There were no missing values for the outcome or predictors in the final model, as noted in the study. Participants were not excluded due to missing data.

DOMAIN 4: Analysis			
		Dev	Val
4.1 Were there a reasonable number of participants with the outcome?		Probably No	n.a.
4.2 Were continuous and categorical predictors handled appropriately?		Yes	n.a.
4.3 Were all enrolled participants included in the analysis?		Yes	n.a.
4.4 Were participants with missing data handled appropriately?		Yes	n.a.
4.5 Was selection of predictors based on univariable analysis avoided?		Probably Yes	
4.6 Were complexities in the data (e.g. censoring, competing risks, sampling of controls) accounted for appropriately?		Yes	n.a.
4.7 Were relevant model performance measures evaluated appropriately?		Yes	n.a.
4.8 Were model overfitting and optimism in model performance accounted for?		Yes	
4.9 Do predictors and their assigned weights in the final model correspond to the results from multivariable analysis?		Yes	
**Risk of bias introduced by the analysis**	**RISK:** *(low/ high/ unclear)*	**Low**	n.a.

Rationale of bias rating:

The risk of bias for this domain is judged to be low. Although the events per variable ratio was below the recommended threshold of 10, which could indicate a potential risk of overfitting (4.1: Probably No), the use of bootstrapping for internal validation helped mitigate this risk (4.8: Yes). Continuous variables were handled appropriately without arbitrary cutoffs (4.2: Yes), and all participants were included in the analysis with no exclusions due to missing data (4.3: Yes; 4.4: Yes). Predictor selection was not solely based on univariable analysis (4.5: Probably Yes), and GEE was used to account for clustering within nursing homes, addressing data complexities (4.6: Yes). Relevant performance measures, including calibration and discrimination, were evaluated properly (4.7: Yes), and predictors in the final model corresponded to the multivariable analysis results (4.9: Yes).

**DOMAIN 4: Analysis**		
**Overall judgement about risk of bias and applicability of the prediction model evaluation**		
**Overall judgement of risk of bias**	**RISK:**<br>*(low/ high/ unclear)*	**Low**
Summary of sources of potential bias:<br>The overall risk of bias is judged as low. Although the EPV was relatively low, bootstrapping was applied to address optimism and potential overfitting. Continuous variables, such as BMI, were handled without arbitrary cutoffs, maintaining the data's integrity. All enrolled participants who met the inclusion criteria were included in the analysis, minimizing exclusion bias. Furthermore, there were no missing data for predictors in the final model, eliminating the need for imputation and reducing bias from missing data. While univariable analysis was initially conducted, predictor selection for the final model relied on multivariable analysis, incorporating clinical relevance and addressing collinearity. The use of GEE accounted for clustering in the hierarchical data structure, reducing bias due to the clustered study design. Model performance was comprehensively assessed through AUC/ROC and calibration, ensuring accuracy. Internal validation with bootstrapping further mitigated overfitting concerns, and predictors and coefficients in the final model were consistent with the multivariable analysis results.		
**Overall judgement of applicability**	**CONCERN:**<br>*(low/ high/ unclear)*	**Low**
Summary of applicability concerns:<br>The applicability concerns are judged to be low. The use of standardized outcome definitions (GLOBIAD and ICD-11) and routine predictors enhances the model's generalizability to similar clinical settings. The study population of long-term care residents aged 65 and older closely aligns with the target population, making the findings broadly applicable in nursing home environments. The outcome assessment approach is feasible under real-life conditions, further supporting the model's applicability.		
